# Fractal Characteristics of Chip Morphology and Tool Wear in High-Speed Turning of Iron-Based Super Alloy

**DOI:** 10.3390/ma13041020

**Published:** 2020-02-24

**Authors:** Xu Zhang, Guangming Zheng, Xiang Cheng, Rufeng Xu, Guoyong Zhao, Yebing Tian

**Affiliations:** School of Mechanical Engineering, Shandong University of Technology, 266 West Xincun Road, Zibo 255000, Chinaxurufeng@sdut.edu.cn (R.X.); zgy709@sdut.edu.cn (G.Z.); tianyb@sdut.edu.cn (Y.T.)

**Keywords:** fractal, chip, tool wear, high-speed turning, iron-based super alloy

## Abstract

Considering that iron-based super alloy is a kind of difficult-to-cut material, it is easy to produce work hardening and serious tool wear during machining. Therefore, this work aims to explore the chip change characteristics and tool wear mechanism during the processing of iron-based super alloy, calculate the fractal dimensions of chip morphology and tool wear morphology, and use fractals to analyze their change trend. Meanwhile, a new cutting tool with a super ZX coating is used for a high-speed dry turning experiment. The results indicate that the morphology of the chip is saw-tooth, and its color changes gradually, due to the oxidation reaction. The main wear mechanisms of the tool involve abrasive wear, adhesive wear, oxidation wear, coating spalling, microcracking and chipping. The fractal dimension of the tool wear surface and chip is increased with the improvement of cutting speed. This work investigates the fractal characteristics of chip morphology and tool wear morphology. The fractal dimension changes regularly with the change of tool wear, which plays an important role in predicting this tool wear. It is also provides some guidance for the efficient processing of an iron-based super alloy.

## 1. Introduction

Iron-based super alloys could maintain good mechanical properties and heat resistance at high temperatures. Therefore, iron-based super alloys had been widely used in aerospace and in the nuclear industry [1]. However, they were classified as hard-to-cut materials, because of the highly corrosive carbide particles present in their microstructure. They also had the characteristics of work hardening, high shear strength and severe tool wear in machining engineering [2]. The processing conditions of super alloys were mainly machined at low-speed, and a large amount of cooling liquid was used. These researches were not in line with the trend of modern machining. Therefore, the high-speed, high-efficiency, high-performance and green processing of super alloys had become a hot spot [3,4].

Mandelbrot proposed the fractal to describe the British coastline length [5]. It was also regarded as an object with self-affine patterns or singularities. Mandelbrot also found that the fractal could be used to analyze irregular graphics, complex broken images, and the relationship between global structural geometry and different scales. In addition, the fractal dimension was a quantitative method to describe irregular phenomena [6]. So far, there was no complete mathematical definition of a fractal. Mandelbrot gave a more extensive and popular concept: the fractal was a form in which the whole and the local had some kind of similarity. There was a property of self-similarity in the structure of fractal graphics. 

Fractal self-similarity referred to the structure and shape of an object, which had great similarity between the whole and the local. In other words, the appearance of the object was similar at different magnification and reduction levels, and the shape of its geometry was also similar. The complexity of the surface shape could be summed up as: the higher the complexity, the greater the fractal dimension. This characteristic could be used to describe the chip morphology and tool wear surface morphology.

At present, the research and application of fractals were mainly focused on cutting force signals, roughness curves and the fractal features of machined surfaces. Fractal theory was used to analyze the cutting force signal, which was obtained during the dressing of carbon fiber composites. It was proven that the proposed fractal index was more effective than its statistical parameters in estimating the tool wear and machined surface quality [7]. At the same time, the cutting force curve and machined surface profile had fractal characteristics. The fractal dimension of the cutting force curve was reduced first, and then increased with the cutting time. The fractal dimension reflected the complexity of the contour curve of the machined surface, and could be used to appraise the quality of the machined surface. The change trend of the fractal dimension of machined surface profile was increased with the cutting speed, but increased firstly, and then reduced with tool wear, and its trend with time was similar to roughness [8,9]. In the sliding wear process, the time series values of the friction force of the graded material and Inconel 718 had an apparent fractal characteristic, and the fractal dimension of the friction forces was decreased gradually [10]. The cutting forces and acoustic emissions signals could be analyzed by fractals while machining Fiber-Reinforced Plastics (FRP). Fractal parameters were efficient for machining quality estimation, and to follow the tool wear evolution [11]. The variation of the fractal also had many associations with wear behavior. The fractal characteristics of the surface topography of Si_3_N_4_ ceramics in rotating ultrasonic grinding by means of quantitative and qualitative methods, were researched. As the spindle speed, cutting depth and feed rate increased, the fractal dimension was firstly increased, and then decreased. The fractal dimension was decreased as the cutting force increased [5]. The fractal dimension of the grinding wheel topography was highly correlated to the wear behavior. The increase of cutting-edge density on the surface of a diamond wheel resulted in the increase of fractal dimension, but an increase in the grain pullout and wheel loading resulted in a decrease in the fractal dimension [12]. The crack extension paths were analyzed by the fractal geometry method. The fractal dimension of crack paths and fracture surfaces were calculated based on the box counting. The results showed that the composites presented higher fractal dimensions [13]. The fractal dimension of a crack was decreased as the distance between the crack and the indentation mark grew larger. The fractal dimension of the indentation-induced crack would be utilized for an effective evaluation of the fracture resistance of the material [14].

Because of the special physical and mechanical properties of super alloys, especially their high strength and low thermal properties, serrated chips were produced in a wide range of cutting speeds. The formation of chips had a significant effect on the change of cutting temperature, cutting force and tool wear. It was crucial to reveal the influence law of the basic factors controlling the chip formation process [15]. In addition, the chip morphology had an important effect on the machined surface quality. Many researchers had focused on the formation mechanism of serrated chips, and had proposed three main theories: adiabatic shear theory, periodic crack theory and the hybrid theory of adiabatic shear-combined ductile fracture [16]. The saw-tooth degree of a chip was increased first and then decreased with the increase of cutting speed, especially when the adiabatic shear band appeared. The microstructure evolution inside the adiabatic shear band also showed significant differences at different cutting speeds and different chip morphologies, which eventually led to changes in the fracture mechanism between chip segments [17]. When dry cutting iron-based super alloys, the chip shape was huge with irregular distribution, which was easy to aggregate, and affected the temperature diffusion [4]. Using the MQCL and MQCL+EP/AW methods, the short spiral fraction form of the chip could be obtained. On the other hand, long, spiral and tangled chips were obtained in the early stage of tool wear in dry machining, which also had some effect upon tool wear. With the increase of tool wear, the chips were shorter spiral shape, and the shape was quite loose in the later stage of tool wear [18]. Although there is no lack of research on chips, most scholars mainly study the shape and formation mechanism of chips, and there are few studies on the fractal characteristics of chips.

Dry cutting processes with high speed, high efficiency and environmental protection had become the trend of development. Therefore, higher requirements for the high resistance of tools had been put forward in the process of dry cutting, and a lot of researches had been studied on the wear forms and wear mechanisms of cutting tools. Previous research and investigations proved that MQL technology could reduce the cutting force, surface roughness and tool wear in cutting to a certain extent, reduced friction and wear during processing, and made it more environmentally friendly [19]. When using the MQL+EP/AW method to process the AISI 1045 carbon steel, the tool wear rate was greatly reduced as compared to the dry cutting. It was found that the friction film formed on the tool surface reduced the friction coefficient, and the active compounds contained in it reduced the adhesion and diffusion rate of the tool wear process, and there were both adhesion wear and a built up edge on the rake face [20]. According to the analysis of tool wear mechanisms, adhesion wear was the main failure mode [21]. The main wear mechanisms of a coated carbide insert during the high-speed machining of iron-based super alloys were adhesive wear and oxidation wear, while that of non-coated inserts were abrasive wear and adhesive wear [1]. The tool failure mechanisms of a ceramic tool, when high-speed machining an iron-based super alloy, included adhesion, chipping, abrasion and notching [22]. A variety of coated tools were used to dry machine high strength steel. It was found that the failure of the tool was mainly caused by flank wear, and the wear mechanism of coated tools also included abrasive wear, adhesive wear, oxidation wear, diffusion wear, spalling off and micro-cracks [23,24]. In the process of high-speed, dry turning of AISI 4340 steel with coated tools, there were some scholars who found that abrasive wear and chipping were the main wear mechanisms [25]. When machining Haynes 282 with coated carbide tools, it was found that the main wear mechanisms of the tool were flank, chipping, built up edge (BUE) and notch wear; the failure of the cutting edge was caused by chipping. The tool life similar to that of lubricant could be obtained under the condition of dry turning, which proved that the application of dry turning in industry was feasible [26,27].

In this work, the test of the high-speed dry turning of iron-based super alloy was carried out, and the SEM images and EDS spectra of the chips and tool wear were analyzed. The fractal theory was used to calculate the fractal dimension, and the fractal features of the chip topographies and the surface textures of the tool wear surface were explored.

## 2. Materials and Methods

### 2.1. Workpiece Material and Tool Material

The workpiece used in the test was iron-based super alloy GH2132 (115 mm diameter × 300 mm long), which was equivalent to A286 in America. Its main physical and mechanical properties, such as the density, tensile strength and yield strength, were 7.916 kg/dm^3^, 930 MPa and 590 MPa, respectively. The hardness of GH2132 after heat treatment was about 36±1 HRC. The chemical composition of GH2132 is listed in Table 1.

The tool holder used in the experiment was MCLNR2020K12. The cutting tool material was the cemented carbide-coated tool produced by the Sumitomo Corporation of Japan, its brand was AC510U, and the model was CNMG120408N-EF. The hardness and flexural force of the tool were 92.6 HRA and 2.6 GPa. The coating type was new super ZX coating. A multi-layer PVD coating film of nanosized TiAlN and AlCrN was used, and the film thickness was 3 μm. The tool nomenclatures are shown in Table 2. 

### 2.2. Cutting Parameters and Method

**Figure 1:**
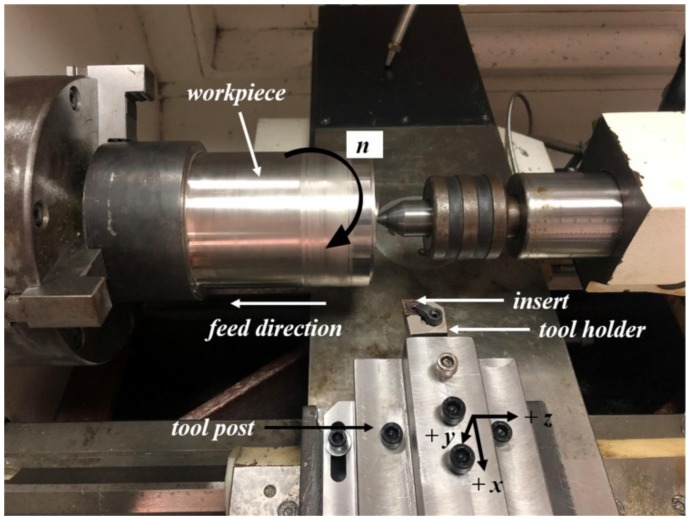
Experimental setup of high-speed dry turning.

The high-speed turning experiment was carried out on the CKD6136i CNC lathe. The experimental setup of high-speed dry turning was shown in Figure 1. The cutting mode was a continuous dry cutting of the outer circle. According to the previous work [28], and information provided by manufacturer, the cutting depth had little effect on tool wear and cutting temperature. Better machining quality and lower cutting force could be obtained under the condition of *a*_p_ = 0.5 mm and *f* = 0.1 mm/r. Therefore, the fixed depth of cut and feed rate were selected. The cutting speed was selected as *v* = 60, 90, 120, 150 and 180 m/min. In order to ensure the reliability of the test, three experiments were conducted at each cutting speed. The average value of the data for each stage was calculated after collecting the data.

In the course of the experiment, the wear of the tool surface was observed by the USB200 digital tool microscope (its maximum magnification was 500×) for each cutting distance (30 mm feed direction). It was measured five times, and then the average was calculated. According to the ISO standard, we took the width VB of the middle wear band of the tool flank face as the evaluation standard. The flank VB = 0.3 mm was selected as the criterion for judging tool failure. To avoid interference, a new insert was used for each experiment. The chips were collected during the experiment. Multiple chips were collected for each parameter, at each cutting stage, to ensure the general applicability of the experiment results. After the test, the chips and the cutting tools were ultrasonically cleaned with absolute ethanol, and dried. Finally, the QUANTA FEG 250 scanning electron microscope (SEM) with energy spectrum analysis (EDS) was used to analyze the tool wear surface and chip microstructure.

### 2.3. Calculation Method of Fractal Dimension

The box dimension method was used to calculate the fractal dimension in this work [9]. Based on the following steps, the SEM image was processed by MATLAB software, and the fractal dimension was estimated by programming. Firstly, the general SEM image had 256 grayscale levels. Secondly, the clearer image could be obtained by enhancing contrast, reducing noise, and so on. Thirdly, a median filtering process was performed, and the image was binarized. In this process, a grayscale image was transformed into a binary image, including pixel values of 0 and 1, black and white. Finally, edge detection and matrix transformation were carried out to detect the position of image feature change. A one-pixel wide edge was produced in the process.

The box counting method was mainly for calculating the fractal dimension of the image. It was assumed that the characteristic length of the basic image was *k* (*k* = 1, 2, 4, 8, …n), and the number of the basic graphic needed to cover the image was *N*. Finally, the fractal dimension *D* was the absolute of the straight line slope which could be obtained by least square method fitting the data points log *k* and log *N*.

## 3. Results and Discussion

### 3.1. Chip Macroscopic Morphology

**Figure 2:**
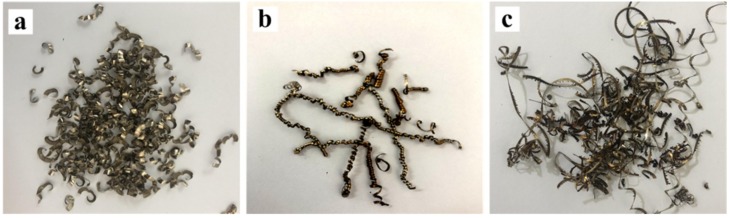
Morphologies of chip in different stages of tool wear under the condition of *v* = 60 m/min, (**a**) VB = 0.04–0.08 mm, (**b**) VB = 0.14–0.18 mm, (**c**) VB = 0.23–0.27 mm.

**Figure 3:**
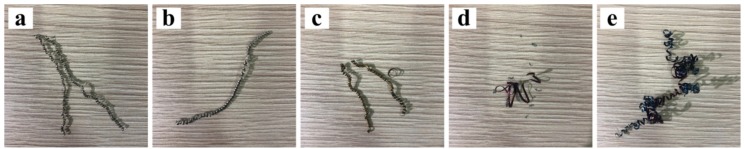
Macroscopic morphologies of chips at *f* = 0.1 mm/r and *a*_p_ = 0.5 mm, (**a**) *v* = 60 m/min, (**b**) *v* = 90 m/min, (**c**) *v* = 120 m/min, (**d**) *v* = 150 m/min, (**e**) *v* = 180 m/min.

The morphologies of the chip in different stages of tool wear under the condition of *v* = 60 m/min are shown in Figure 2. It can be seen that in the early stage of tool wear, the morphology of the chip is mostly fragmented. Subsequently, in the middle stage of wear, the chip gradually becomes a curly shape. Finally, in the later stage of tool wear, the shape of the chip is continuous and wound together. This is due to the fact that with the tool wear, the tool rake and flank surface bonding and oxidation diffusion make the blade and the rake surface wear seriously, and the crimping groove angle changes, resulting in the chip flow through the tool surface resistance increases, chip breaking mode changes. The macroscopic morphologies of the chips are shown in Figure 3. Because of the chips flute structure of the tool, the chips are relatively regular, and mainly appear curled. As the cutting speed increases, the color of the chip gradually changes from golden yellow at the beginning to dark yellow to blue-violet obviously. This indicates that the cutting temperature gradually increases (the temperature ranges from about 703 K to 950 K [29]), and the chip may undergo an oxidation reaction, resulting in a change in the color of the chip.

### 3.2. Chip Microscopic Morphology

**Figure 4:**
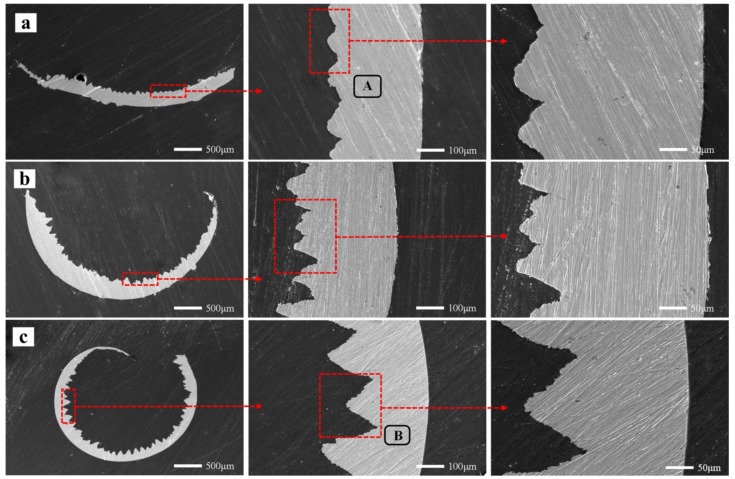
Chip microscopic images with different multiples at *f* = 0.1 mm/r and *a*_p_ = 0.5 mm, (**a**) *v* = 60 m/min, (**b**) *v* = 120 m/min, (**c**) *v* = 180 m/min.

Figure 4 shows the chip microscopic images with different multiples. When the cutting speed is low (Figure 4a), there are a few saw-tooth contours at the top of the chip, and the chip morphology is more zonal and irregular. In the process of high-speed or even higher speed cutting, there is a relatively regular triangular saw-tooth at the top of chip section, which is called the saw-tooth chip. It is caused by the instability of thermoplastic shear due to the intense and concentrated local shear in the first deformation region. Thermoplastic shear instability is influenced by shear strain, deformation temperature and shear strain rate. When the deformation temperature is constant, the critical shear strain of the critical shear strain rate in the critical state is inversely proportional. Therefore, the higher the deformation temperature, the larger the critical shear strain or shear strain rate required for the thermoplastic shear instability. Materials with poor thermal characteristics, such as iron-based super alloys, are more susceptible to thermoplastic shear instability. On the other side of the saw-tooth is a relatively smooth band. Observing the overall variation trend of chip morphology, it is found that with the increment of cutting speed (Figure 4b), the top of the chip has more obvious, regular and dense saw-tooth distributions. Additionally, in high-speed cutting conditions (Figure 4c), each saw-tooth on both sides will also have a smaller serrated formation. Thus, the saw-tooth degree of the chip will be increased with the increase of speed.

**Figure 5:**
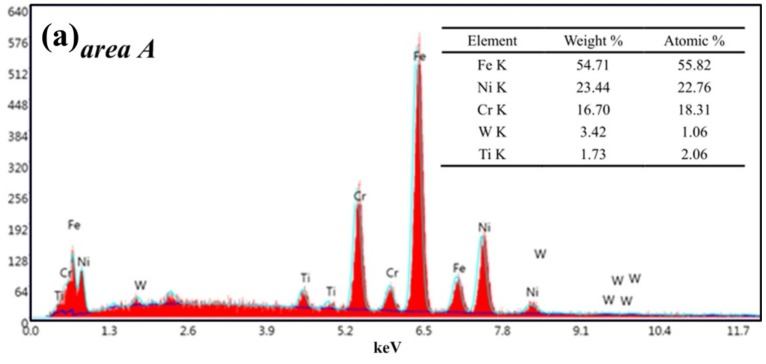

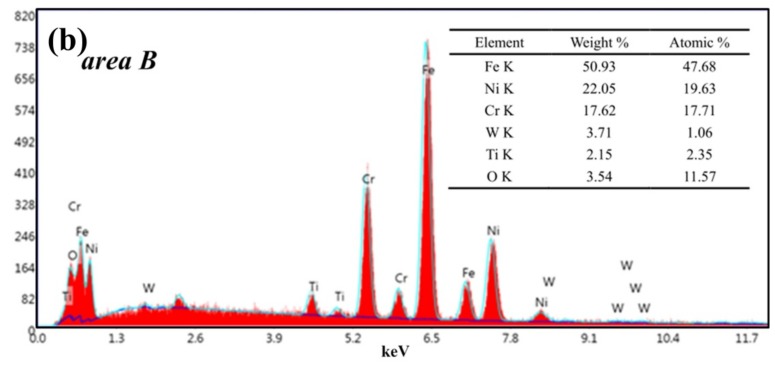
Analysis of chips energy-dispersive X-ray spectrum (XDS), (**a**) area A in Figure 4a, (**b**) area B in Figure 4c.

Observing the analysis of the chips EDS spectrum in Figure 5, it is found that under low-speed cutting conditions (*v* = 60 m/min), the elements in the chips are mainly Fe, Ni, Cr and a small amount of W, Ti (Figure 5a). The contents of W and Ti are very low. The workpiece also contains 2%–2.3% of Ti. Therefore, the source of the Ti element is likely to come from the workpiece itself, and there may be a small amount from the coating. The content of W is only 3%–4%. Therefore, we speculate that this small amount of the W element may come from the tool matrix material in the coating peeling area. 

So it can be seen that in addition to the workpiece material, the chips also take away part of the tool base and coating material. This is due to the mechanical stress and the cutting heat received during the cutting process. The rake face, the cutting edge of the tool wear, and the chip groove angle are changed slightly. Because of the resistance of the chips flowing through the surface of the tool increase, the contact time between chip and tool is increased. Therefore, more coating elements and a small number of tool matrix elements may be adhered or diffused into the chip.

After entering the high speed condition (*v* = 180 m/min), it is found that a small amount of O elements existed in the chips (Figure 5b). It can be seen that the cutting temperature become higher at this speed, and the air enters the cutting area during the cutting process. The oxidation diffusion reaction occurs with the material elements in the cutting tool and workpiece. The results of this analysis are consistent with the analysis of chip color change in the macroscopic morphologies. It can also be inferred that the tool wear and oxidation wear occurred during the cutting process.

**Figure 6:**
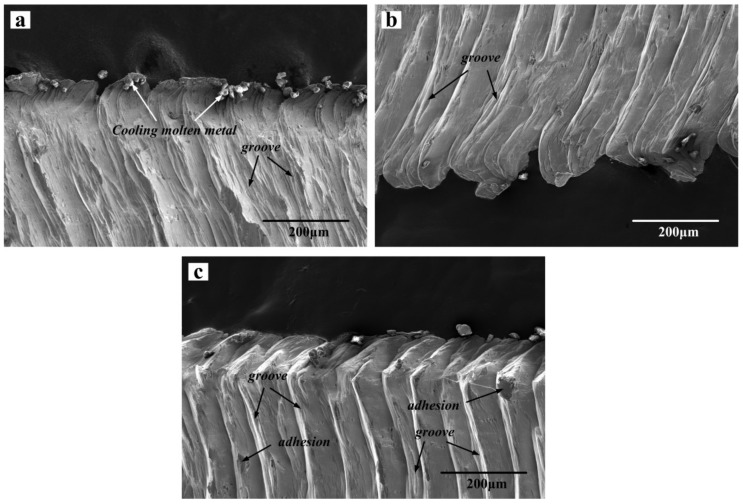
Scanning electron microscopy (SEM) images of the front morphology of the chip at three different cutting speeds, (**a**) 60 m/min, (**b**) 120 m/min, (**c**) 180 m/min.

Figure 6 shows the SEM images of the front morphology of the chip at three different cutting speeds, where (a), (b) and (c) are the frontal morphology at cutting speeds 60, 120 and 180 m/min, respectively. It can be seen from the figure that the uneven grooves are distributed on the front of the chips. As the cutting speed increases, the grooves are also slowly squeezed to the tip of the saw-tooth shape, and there is a small amount of adhesion. As well as this, at the burrs of the chips, there are a large number of cooling metal particles, which may be due to the falling of the build-up edges formed by the tool and bonding to the chips. It can also be seen from the changes in the frontal morphology that the saw-tooth morphology of the chips becomes more and more obvious as the cutting speed increases. As the cutting speed increases, the shear force increases sharply on the shear slip surface, the shear deformation tends to be concentrated gradually, and the deformation tends to be uneven gradually, which leads to the more clear and obvious slip on the shear surface, and so the generation of the saw-tooth is also more and more obvious. On the other hand, the increase of processing speed leads to the same increase of processing temperature, and the thermal conductivity of iron based alloy is extremely low, so the heat emission in the process is very slow, resulting in a large amount of heat accumulated in the region of the processing range, which causes the stability of the shear deformation to be unstable. Therefore, the chip will change from a strip-like morphology to a zigzag morphology.

With the continuous improvement of processing speed, the material of the workpiece will appear more and more strain rate, compared with the lower speed. In addition, with the continuous increase of processing speed, the material portion that the tool cuts off at the same cutting time will increase sharply, so more and more energy is required to form more and more heat. More and more processing heat will not be lost in the future, which is mainly due to the sharp decrease the time when this part of chip produces deformation when the processing speed increases. These phenomena will promote the occurrence of adiabatic shearing in the chip, resulting in the increase of the deformation capacity of the chip. Therefore, the continuous increase of processing speed is a very beneficial factor for the generation of saw-tooth chips.

### 3.3. Fractal Analysis of Chip Morphology

**Figure 7:**
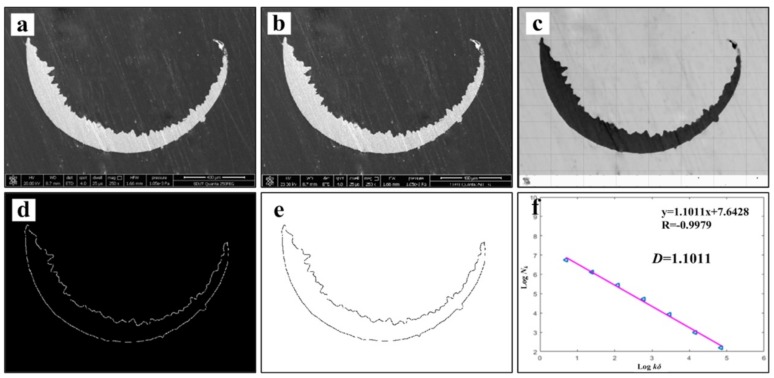
Chip morphology image and its fractal dimension at *v* = 60 m/min, (**a**) SEM image of chip, (**b**) grayscale processing, (**c**) median filter processing, (**d**) edge feature extraction, (**e**) edge detection and (**f**) linear fitting.

**Figure 8:**
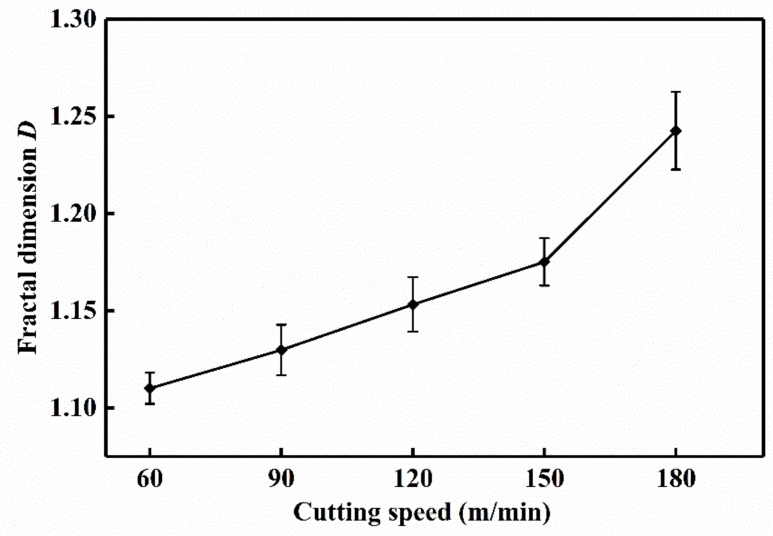
Chip fractal dimension variation curve at *f* = 0.1 mm/r and *a*_p_ = 0.5 mm.

Figure 7 shows the chip morphology image and its fractal dimension at *v* = 60 m/min. The main processes are grayscale processing, median filter processing, edge feature extraction, edge detection and linear fitting [13,30]. In order to avoid the contingency of the experiment, three small pieces of chip are selected as the calculation object at each speed, and finally the average value is taken to draw the change curve. The chip fractal dimension variation curve is exhibited in Figure 8. It can be found that with the increase of cutting speed, the proportion of details reflected in the microscopic morphology of the chip is gradually increasing, and the fractal dimension of chip becomes larger and larger. It is speculated that with the increase of cutting speed, the tool wear will be intensified. The adhesive wear, abrasive wear and even microchipping, occur at the cutting edge, make the micro-morphologies of chip become more complicated, then the quality of the machined surface is reduced, and the roughness will be increased gradually. As a result, the edge of saw-teeth on both sides of the chip is increased, and the fractal dimension is increased under microscopic morphology. At the same time, chips can also counteract on the cutting tool and workpiece, which accelerate the tool wear and reduce the machining quality of the workpiece surface. When the cutting speed increases from 150 to 180 m/min, the fractal dimension of the chip has an obvious exponential increase, with a slope greater than the previous several velocities. This may be due to the qualitative change of the saw-tooth shape of the chip in this speed range as the cutting speed increases. Its jagged shape is more dense and regular, and the microscopic details suddenly increase, which leads to the sudden increase of fractal dimension. According to the variation of fractal dimension, the saw-tooth morphology of the chips obtained at this cutting speed is more regular and standard.

Therefore, understanding the mechanism and characteristics of chip formation, calculating and analyzing the fractal dimension of chip contour, and observing its changing trend, can effectively understand the details of chip morphology and texture changes. We can reasonably predict the chip change in the cutting process with the help of fractal theory, and use it to estimate the degree of tool wear approximately.

### 3.4. Tool Wear Mechanism

#### 3.4.1. Macro Morphology Analysis of Tool

**Figure 9:**
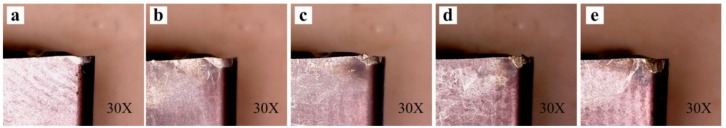
Macroscopic morphology of the tool wear at different cutting speeds, (**a**) *v* = 60 m/min, (**b**) *v* = 90 m/min, (**c**) *v* = 120 m/min, (**d**) *v* = 150 m/min, (**e**) *v* = 180 m/min.

The macroscopic morphology of the tool wear at different cutting speeds is shown in Figure 9. As can be seen from the whole, with the increase of the cutting speed, the tool flank wear becomes more and more serious. At the low and middle cutting speeds, the wear area is also smooth and bright. But when the high-speed cutting is entered, the wear of the tool becomes severe. The wear area becomes more rough and irregular, the obvious adhesion and chipping phenomenon is found, and the color darkens gradually, which may be due to the oxidation reaction in the cutting process with the increase of the speed, where the wear part of the tool tip gradually tends to be dark yellow.

**Figure 10:**
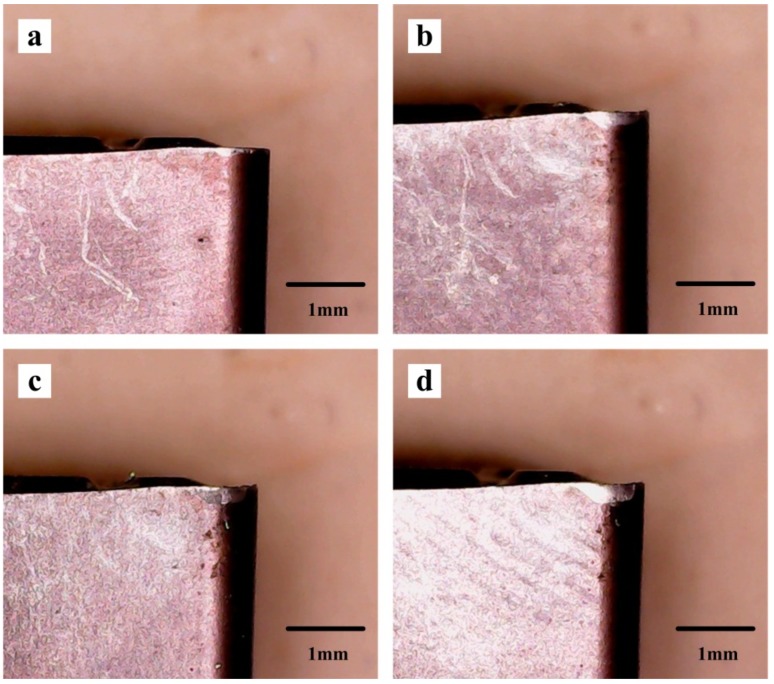
Macroscopic morphology of the tool flank face wear process at *v* = 60 m/min, *f* = 0.1 mm/r and *a*_p_ = 0.5 mm, (**a**) t = 2.45 min VB = 0.097 mm, (**b**) t = 9.26 min VB = 0.147 mm, (**c**) t = 15.3 min VB = 0.175 mm, (**d**) t = 26.8 min VB = 0.309 mm.

Figure 10 shows the macroscopic morphology of the tool flank face wear process at *v* = 60 m/min, *f* = 0.1 mm/r and *a*_p_ = 0.5 mm. As can be seen from the Figure 10a, the cutting tool wear speed is faster at the beginning of the cutting period, and the amount of the flank face wear has been close to 0.1 mm in less than three minutes. This is due to the larger surface roughness and microdefects of new sharpened tools (such as microcracks, oxidation or decarburization), the actual contact area between the tool and the workpiece is small, and the contact point bonding is serious, so the tool wear rate is high. After the initial wear, the rough surface of the tool is gradually flattened, and enters a stable wear stage [9]. The tool wear state at this stage is slow and uniformly stable (Figure 10b,c) and the wear width increases approximately proportionally slowly as the cutting time increases. During the period of sharp wear, the wear rate of the tool increases sharply, and the life of the tool reaches its limit in about 27 min (Figure 10d). The cutting edge of the flank face has obvious scratches and hard particles, and the microchipping phenomenon at the tip of the blade will cause the tool to be damaged and lose its cutting ability.

#### 3.4.2. Micro Morphology Analysis of Tool

**Figure 11:**
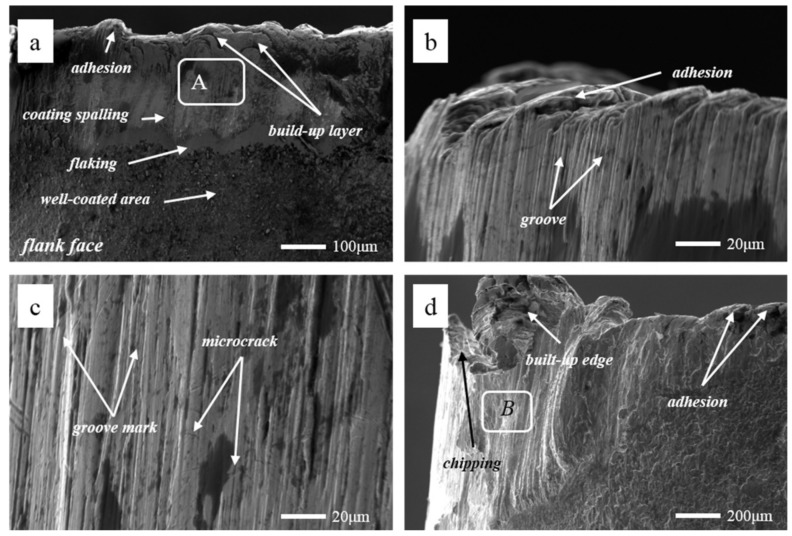
Tool wear microscopic morphologies at *f* = 0.1 mm/r and *a*_p_ = 0.5 mm, (**a**) flank face wear area at *v* = 60 m/min, (**b**) wear profile of the tool tip at *v* = 60 m/min, (**c**) wear area below the tool tip at *v* = 60 m/min, (**d**) tip wear morphology at *v* = 180 m/min.

**Figure 12:**
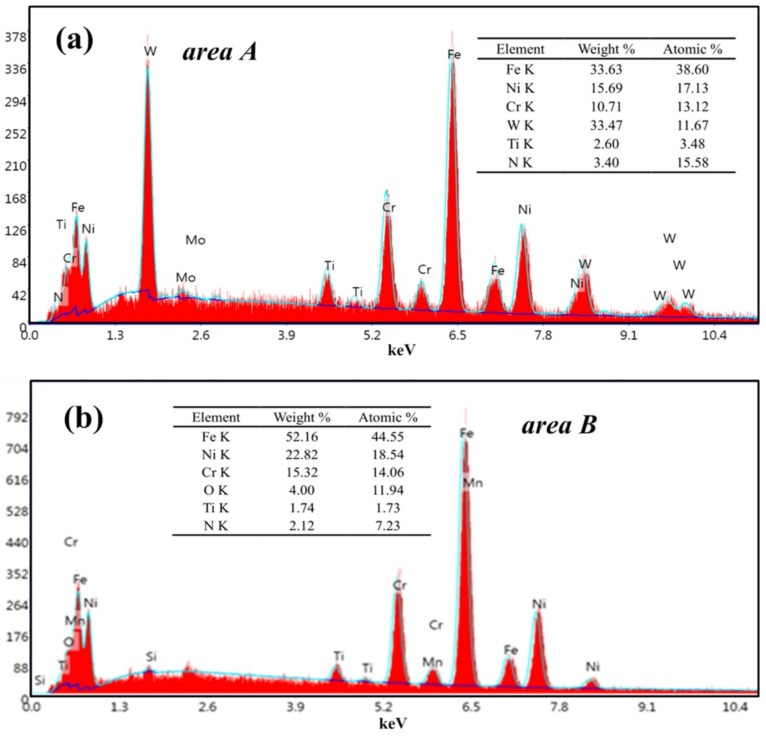
EDS energy spectrum analysis, (**a**) area A in Figure 9a, (**b**) area B in Figure 9d.

Tool wear microscopic morphologies are shown in Figure 11. Figure 11a shows the flank face wear area at *v* = 60 m/min. The phenomena of coating flaking and coating spalling on the tool surface are observed obviously, and there are some adhesions found on the cutting edge. At the same time, it can be seen that there are a lot of lamellar buildup layers in the wear area below the cutting edge, which indicate that the main wear mechanism of the cutting tool is adhesion wear. Some scratches in the spalling area of the coating may be due to when the chip in formation is extruded between tool and workpiece surface, and some adheres to the tool flank face. In the process of adhesion and shedding, it may lead to the formation of scratches. The EDS energy spectrum analysis (Figure 12a) of the selected area A in Figure 11a reveals that the main chemical elements in this part are W, Fe, Ni and Cr, which are tool matrix materials and workpiece materials, and have a small amount of N and Ti. It is confirmed that there are adhesions of the workpiece material in this area, and the exposed area exposes the tool base. At the same time, Ni and Cr elements reduce the hardness and cutting performance of the tool on the surface of the tool, increase the affinity of the workpiece and the tool, and accelerate the adhesive wear. O elements are not found in the energy spectrum analysis, and it is inferred that there is no oxidation wear on the tool surface at this cutting speed.

Figure 11b shows the tool tip wear profile at *v* = 60 m/min. It can be observed that there is a number of neat grooves below the cutting edge, and a small number of small pieces of adhesion on the cutting edge (the rake face). This is because the tool at the end of the wear is worn more strongly, and the tool is subjected to more force and heat. In the end of the cutting, the wear width of the flank face is observed by a digital microscope, and the rate of the tool wear is found to be fast. At the same time, there are a large number of sparks splashing, which are observed in the cutting position of the tool, and the vibration of the machine tool is more serious, which are due to the greater force and heat produced by the cutting.

Wear area below the tool tip at *v* = 60 m/min is shown in Figure 11c. It is observed that there are obvious fine groove marks and brittle fractures, such as microcracks. It may be due to the friction of the cemented carbide particles in the workpiece during the cutting process, which has a significant scribing effect on the tool and produces abrasive wear. Figure 11d exhibits the tip wear morphology at *v* = 180 m/min. The wear of the tool is mainly concentrated on the tip end and the flank near the tip. There is a very large amount of built-up edge above the cutting edge and a phenomenon of chipping. The flank has obvious block, buildup adhesion layer and coating wear. The adhesion of the built-up edge on the rake face can effectively reduce the friction and contact between the cutting edge and the high-speed flowing chips, reducing the chip deformation, along with the cutting temperature and cutting force. Moreover, the diffusion of chips and workpiece elements to the tool is reduced. Although the built-up edge can replace cutting edge cutting and protect the cutting edge to a certain extent, the accumulated blunt circular arc edge results in extrusion or overcutting, which reduces the machining accuracy. At the same time, during high speed cutting, it is very easy to fall off or break locally under the action of external force or vibration, and then it is produced and shedding over and over again. This phenomenon reduces the adhesion between the coating and the tool, makes it easier to cause the phenomenon of blade collapse, and reduces the tool life. The EDS energy spectrum analysis (Figure 12b) of area B in Figure 11d shows that the main elements are Fe, Ni, Cr, and a small amount of O elements exist. 

It can be seen that the cutting temperature is higher at the higher cutting speed, air enters the cutting area, and the oxidation reaction occurs with parts of the workpiece and the tool.

### 3.5. Fractal Analysis of Tool Wear

**Figure 13:**
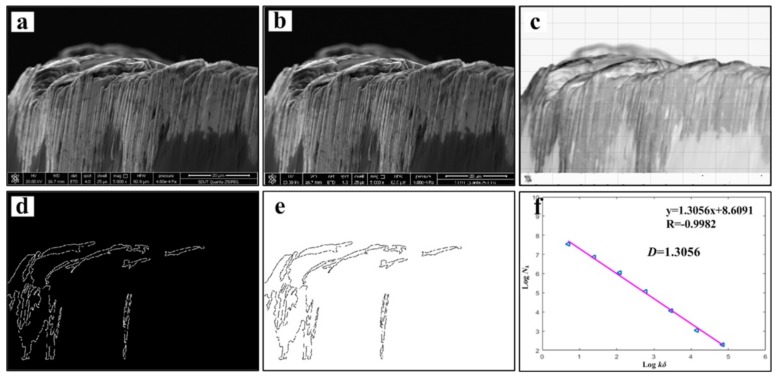
Tool wear image and its fractal dimension at *v* = 60 m/min, (**a**) SEM image of tool wear, (**b**) grayscale processing, (**c**) median filter processing, (**d**) edge feature extraction, (**e**) edge detection and (**f**) linear fitting.

**Figure 14:**
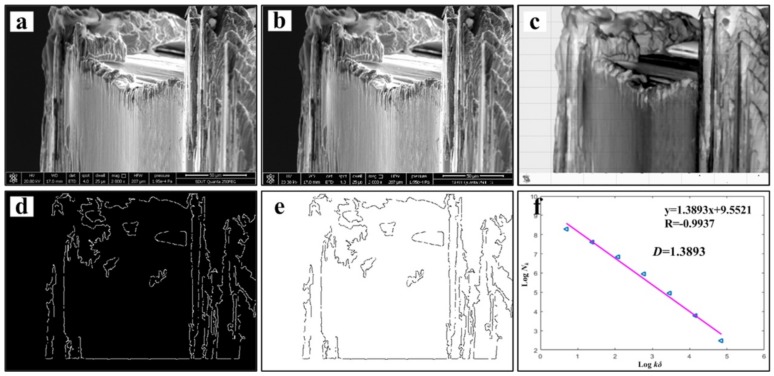
Tool wear image and its fractal dimension at *v* = 180 m/min, (**a**) SEM image of tool wear, (**b**) grayscale processing, (**c**) median filter processing, (**d**) edge feature extraction, (**e**) edge detection and (**f**) linear fitting.

**Figure 15:**
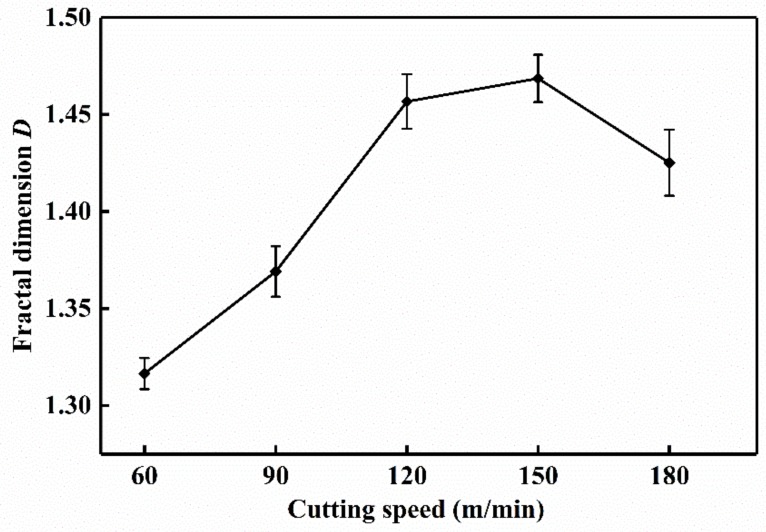
Tool wear fractal dimension variation curve at *f* = 0.1 mm/r and *a*_p_ = 0.5 mm.

Figure 13 and Figure 14 show the tool wear image and its fractal dimension at *v* = 60 m/min and *v* = 180 m/min mainly include the SEM image of tool wear, grayscale processing, median filter processing, edge feature extraction, edge detection and linear fitting [13,30]. Select the tool wear image when the tool flank face wear width is close to 0.3 mm, in order to reduce the contingency, three images are selected at each cutting speed. Take the average of three data after calculating the fractal dimension of each sheet. The tool wear fractal dimension variation curve is exhibited in Figure 15. As the cutting speed increases, the fractal dimension of the tool surface is gradually increasing. Due to cutting speeds becoming higher, the friction between the workpiece and the tool becomes more and more intense. Abrasive wear and adhesion wear on the surface of the tool are getting more serious. As a result, the texture details and fractal dimension of the tool surface are increased gradually. Under the condition of higher cutting speeds (150–180 m/min), the tool may have a large area of coating spalling and tip breaking, due to the influence of greater force and higher heat. The tool coatings and the base materials are taken away, and the surface texture of the exposed tool base surface is rather regular and smooth. So the fractal dimension becomes smaller.

By comparing the fractal dimension of the chip morphology and tool wear image, it is found that the fractal dimension of chip is lower than that of the tool wear surface at different cutting speeds. This is because fractal dimensions represent how much space is occupied, representing the complexity of the image. Both the detail and complexity of the tool wear surface image are relatively larger than that of the chip image. Therefore, in the process of calculating the fractal dimension of the image, the detail after the edge feature extraction of the chip image is obviously less than that of the tool wear image. The fractal dimension of the chip is between 1.1–1.23, and the fractal dimension of the tool is in the range of 1.3–1.46.

The fractal dimensions of tool wear morphology and chip morphology can show the tool wear status to a certain extent. Using fractal theory, by calculating the fractal dimensions of tool wear morphology and chip profile, we can discuss the chip formation mechanism, tool wear morphology, and changes in wear mechanism. The change of fractal dimension is used to roughly predict the tool life and wear mechanism under different parameters, providing a certain theoretical guidance for future efficient machining.

## 4. Conclusions

In this work, the high-speed dry turning of iron-based super alloy was carried out by using coated cemented carbide tools. Through the analysis of chip morphology and tool wear, the fractal features of the chip shape and tool wear change were explored, and the wear mechanisms of the tool were analyzed. However, we only explored the test results under dry cutting conditions, and did not use cutting fluid for the test, and only explored the fractal characteristic of a coated cemented carbide tool after machining. The applicability of the method for other types of tools (ceramic tools, CBN tools, etc.) remains to be explored in the future. Further validation is needed to make it a viable option for the manufacturing industry. The main conclusions of this work were as follows.

- The morphology of the chip and tool wear surface had fractal characteristics. Moreover, the fractal dimension was changed regularly with tool wear, which can be used to predict tool wear to a certain extent.
- The profile of the chip was saw-tooth caused by thermoplastic shear instability. The chip color changed due to the oxidation diffusion reaction, and the saw-tooth and fractal dimension of the chip was increased with the increment of cutting speed.
- The main wear forms of the coated tool were the normal rake wear and flank wear, and abnormal wear, such as spalling and chipping. It was characterized by the generation of the buildup edge on the rake face, secondary buildup layers and bond block on the flank face, the grooves, scratches and microcracks at the edge of the cutting lip. The main wear mechanisms involved abrasive wear, adhesive wear and oxidative wear.
- The fractal dimension of the tool wear surface was increased with the increase of cutting speed, while it was decreased at high speed due to large-area coating spalling and chipping. The fractal dimension of the tool wear surface was larger than that of chip, because the former had more details in the image.

## Figures and Tables

**Table 1:** Chemical composition of GH2132 (wt %).

**C**	**Cr**	**Ni**	**Mo**	**Ti**	**Fe**	**V**
≤0.08	14.0–16.0	23.0–25.0	0.5–1.0	2.0–2.3	Bal	0.1–0.7
**B**	**Mn**	**Al**	**Si**	**P**	**S**	
≤0.01	≤2.00	≤0.40	≤0.50	≤0.03	≤0.02	

**Table 2:** Tool nomenclatures.

Rake Angle *γ* (°)	Clearance Angle *α* (°)	Inclination Angle *λ* (°)	Nose Radius *r* (mm)
6	−6	0	0.8
